# Indigenous Negative-Pressure Wound Therapy vs Conventional Dressing for Abdominal Wound Dehiscence in a Resource-Limited Setting

**DOI:** 10.7759/cureus.113800

**Published:** 2026-08-02

**Authors:** Priyanka Kerketta, Arunav Roy, Prabha R Lakra, Ritvik Prakash, Jyoti Kumari

**Affiliations:** 1 Plastic Surgery, Rajendra Institute of Medical Sciences, Ranchi, IND; 2 Anesthesiology, Rajendra Institute of Medical Sciences, Ranchi, IND

**Keywords:** abdominal wound dehiscence, low-cost npwt, negative-pressure wound therapy, open abdomen, resource-limited settings, vacuum-assisted closure

## Abstract

Background and objective

The open abdomen and postoperative abdominal wound dehiscence are challenging surgical problems, and negative-pressure wound therapy (NPWT) is the preferred method of temporary abdominal closure. The high cost of commercial NPWT systems frequently limits their accessibility, especially in low- and middle-income countries, prompting the use of indigenous, low-cost alternatives whose comparative performance remains poorly documented. The objective of this study was to compare the effectiveness of an indigenous NPWT (vacuum-assisted closure) system with conventional saline-gauze dressing in the management of postoperative abdominal wound dehiscence.

Materials and methods

In this prospective, single-center, comparative study conducted at Rajendra Institute of Medical Sciences, Ranchi, India, from January to September 2024, 66 adults (≥18 years) with postoperative abdominal wound dehiscence following midline laparotomy or an open abdomen were managed with either indigenous NPWT (n = 31) or conventional saline-gauze dressing (n = 35). The indigenous system used a locally assembled, autoclaved open-cell foam with a 16-Fr tube and an adhesive polyurethane drape connected to wall suction at 120 mmHg. Outcomes were time to healthy granulation, number of dressing changes, percentage wound-area reduction at one week, rate of granulation-tissue formation, pain (Visual Analog Scale at 24 hours, 48 hours, and seven days), need for additional debridement, and complications. Between-group comparisons were done using the independent t-test or the Mann-Whitney U test for continuous variables, whereas categorical variables were analyzed with the chi-square test or Fisher’s exact test. A p-value <0.05 was defined as statistically significant.

Results

The groups were comparable at baseline in age, sex, and wound area (all p > 0.05). Compared with conventional dressing, indigenous NPWT was associated with a shorter time to healthy granulation (9.84 ± 1.19 vs 16.46 ± 4.20 days; p < 0.001), less frequent dressing changes (2.10 ± 0.30 vs 17.80 ± 4.69; p < 0.001), greater wound-area reduction at one week (54.2% ± 10.3% vs 42.7% ± 14.9%; p < 0.001), and a faster rate of granulation-tissue formation (2.50 ± 0.58 vs 1.00 ± 0.18 cm²/day; p < 0.001). Pain scores were consistently lower in the NPWT group at 24 hours (3.81 ± 0.75 vs 6.03 ± 0.79; p < 0.001) and throughout follow-up. Fewer patients required additional debridement (7/31, 22.6% vs 22/35, 62.9%; p = 0.001). Complications were infrequent (5/66, 7.6%) and did not differ significantly between groups (2/31, 6.5% vs 3/35, 8.6%; p = 1.00).

Conclusions

Indigenous NPWT was associated with faster granulation and improvement in early wound-healing parameters, fewer dressing changes, greater early wound-area reduction, and less pain than conventional saline-gauze dressing in postoperative abdominal wound dehiscence. As a low-cost, locally assembled alternative, it offers a practical option for resource-limited settings. The nonrandomized, single-center design warrants confirmation in randomized controlled trials.

## Introduction

The management of the open abdomen has evolved substantially over the past 20 years, particularly in the context of trauma, sepsis, and abdominal compartment syndrome. Once regarded as a last resort, the open abdomen is now a deliberate, staged component of surgical strategy, especially in damage-control surgery, where its appropriate use reduces intra-abdominal hypertension, aids physiological stabilization, and improves survival in critical surgical patients [1,2].

Temporary abdominal closure (TAC) is a fundamental component of open-abdomen management. Its objectives include safeguarding the intra-abdominal viscera, minimizing fluid and heat loss, decreasing the risk of injury to the bowel and subsequent formation of fistula, and enabling delayed fascial closure. Available approaches include simple sterile-towel packaging, the Bogota bag, fascial traction via mesh, and negative-pressure wound therapy (NPWT) [3]. Simpler methods are readily available but are associated with prolonged hospital stays, persistent intra-abdominal infection, fascial retraction, delayed healing, and higher rates of enteroatmospheric fistula, and primary fascial closure after their prolonged use can be difficult [4-6].

Applied directly to the wound bed, NPWT delivers subatmospheric pressure that promotes granulation tissue formation, reduces edema, enhances tissue perfusion, facilitates exudate removal, and preserves fascial planes, thereby increasing the likelihood of successful delayed primary closure [7,8]. Systematic reviews report that NPWT-based techniques achieve among the lowest associated mortality and highest fascial-closure rates [7,9], and the World Society of the Abdominal Compartment Syndrome has recommended NPWT as the preferred modality for managing the open abdomen [10].

Despite these advantages, commercial NPWT systems remain prohibitively expensive in many settings, and supply limitations restrict their use in resource-limited and government institutions, especially in low- and middle-income countries (LMICs) [11]. To address this, surgical teams have developed indigenous NPWT systems from readily available materials, such as open-cell foam, suction tubing, an airtight drape, and wall suction, to reproduce the principles of negative-pressure therapy at a fraction of the cost [11,12]. Reports suggest such systems achieve wound-healing outcomes comparable to commercial devices in infected wounds and are a viable option for open-abdomen management in constrained settings [12,13].

Structured evidence on the performance of these devices in routine institutional practice nonetheless remains limited. In our institution, an indigenous NPWT system is routinely used for TAC, but its efficacy, complication profile, and feasibility have not yet been formally evaluated. The aim of this study was to assess the effectiveness of indigenous NPWT compared with conventional saline-gauze dressing in the management of postoperative abdominal wound dehiscence. The primary objective was to compare the time to appearance of healthy granulation tissue and disappearance of slough between the two groups; secondary objectives were to compare the percentage reduction in wound size at one week, the rate of granulation-tissue formation (cm²/day), and pain (Visual Analog Scale, VAS) at 24 hours, 48 hours, and seven days.

## Materials and methods

Study design and setting

This was a single-center, prospective, comparative study conducted in the Department of Surgery, Rajendra Institute of Medical Sciences, Ranchi, India, between January and September 2024. Reporting follows the STrengthening the Reporting of OBservational studies in Epidemiology (STROBE) guidance for observational studies. Patients were managed across two surgical units, whose established dressing practice determined treatment allocation.

Ethical considerations

The study was approved by the Institutional Ethics Committee (ECR/769/INST/JH/2015/RR-21). Written informed consent for treatment and for open-access publication was obtained from all participants. No identifying patient information is included in this article.

Participants and eligibility

Consecutive patients who developed postoperative wound dehiscence (complete or incomplete) after midline abdominal surgery, or in whom the abdomen was left open after surgery, were screened. Inclusion criteria were age ≥18 years and dehiscence as defined below. Patients were excluded if they had a true open abdomen with freely exposed viscera, surgically created stomas, an entero-cutaneous fistula, abdominal tuberculosis, or severe hypoalbuminemia or anemia, or were receiving corticosteroids, immunosuppressive agents, or chemotherapy, or had a known immunodeficiency. All included patients had wound dehiscence with a granulating wound bed; none had freely exposed viscera.

Sample size

Sample size estimation was based on detecting a difference in the mean wound-healing time between two independent groups. Using the formula for comparing two means with a two-sided significance level of 5% (Zα/2 = 1.96) and 90% power (Zβ = 1.28), a conservative expected mean difference of six days, and a pooled SD of 5.79 days [14], the required sample was approximately 20 patients per group. Consecutive eligible patients presenting during the study period were enrolled, representing a nonprobability consecutive sampling approach, and a total of 66 patients were included (31 NPWT, 35 conventional dressing). Allocation to the indigenous NPWT or the conventional saline-gauze dressing arm was not randomized; each patient received the wound-care modality routinely practiced by the admitting surgical unit, and consecutive eligible patients were assigned to the corresponding arm accordingly.

Treatment groups

Indigenous NPWT

After initial debridement, a locally purchased open-cell foam pad was steam-autoclaved and sculpted to the wound dimensions. A tunnel was created within the foam, and a 16-Fr tube was inserted. The wound was sealed with a 30 × 30 cm polyurethane drape to establish an airtight environment, after which continuous negative pressure of 120 mmHg was delivered through the drainage tube using the wall-mounted suction system, with exudate collected in an inline canister. In this cohort, foam was applied to the granulating wound bed only; no patient had freely exposed viscera. The dressing was first inspected on day 5, then on day 8, and subsequently as indicated.

Conventional Dressing

After initial debridement, dressings were performed under aseptic conditions using sterile gauze soaked in normal saline and changed once daily.

For both groups, blood glucose was monitored and controlled with insulin as required; hemoglobin and serum albumin were assessed weekly and corrected as needed. Wound-base cultures were obtained every three days; a standard empirical antibiotic regimen was started and subsequently guided by culture sensitivity. Analgesia was administered in both groups according to the World Health Organization analgesic ladder. Additional debridement was performed whenever slough was visible on inspection by the treating surgeon. Dressings were continued until the wound was fit for secondary suturing or up to eight weeks. Secondary suturing was performed once the wound was free of pus and slough with healthy granulation tissue.

Outcomes and operational definitions

Wound dehiscence was defined as the postoperatively unintended separation of part or all layers of the abdominal-wall incision, with or without exposure of abdominal contents [15]. Healthy granulation tissue was defined as red, moist, well-vascularized tissue in the wound bed on clinical assessment, and slough as yellow or white necrotic tissue [16]. Severe hypoalbuminemia was defined as serum albumin <3 g/dL [17]. Intensity of pain was evaluated using a 10-point VAS, where 0 represented no pain, and the worst pain imaginable was indicated by 10 [18]. Percentage reduction in wound size was calculated as follows [19]:

\(\text{Percentage wound-area reduction} = \frac{\text{Initial wound area} - \text{Wound area at day 7}} {\text{Initial wound area}} \times 100\)

The rate of granulation-tissue formation was calculated as follows:

\(\text{Rate of granulation-tissue formation} = \frac{\text{Granulated area (cm}^2\text{)}}{\text{Number of days since initiation of therapy}}\)

Wound area was estimated as the product of the maximal length and breadth measured with a sterile ruler at each dressing change; serial wounds were also documented photographically. The need for additional debridement and any complications (bleeding, infection, and maceration) were recorded.

Statistical analysis

Continuous data are presented as mean ± SD and categorical data as frequencies and percentages. Distribution normality was evaluated using the Shapiro-Wilk test and equality of variances using Levene’s test. Depending on data distribution, comparisons of continuous variables between groups were performed using either the Welch independent-samples t-test or the Mann-Whitney U test. Categorical variables were analyzed with the Pearson chi-square test or Fisher’s exact test, as appropriate. All statistical tests were two-sided, with p < 0.05 considered indicative of statistical significance. Statistical analyses were conducted using jamovi version 2.6.44 (The jamovi Project, Sydney, Australia). There were no missing data; all 66 enrolled patients completed the planned follow-up and were included in the analysis.

## Results

Baseline characteristics

Sixty-six patients were included, 31 managed with indigenous NPWT and 35 with conventional dressing. The mean age was 44.55 ± 17.31 years (range 18-84), and 46 patients (69.7%) were male. At baseline, the two groups were well matched with respect to age, sex, emergency presentation, and initial wound area (all p > 0.05), indicating balanced groups despite nonrandomized allocation (Table 1).

**Table 1:** Baseline demographic and clinical characteristics by treatment group. Continuous variables were analyzed using the Mann-Whitney U test, while categorical variables were compared with the Pearson chi-square test. NPWT, negative-pressure wound therapy

Characteristic	Indigenous NPWT (n = 31)	Conventional (n = 35)	Test statistic	p-value
Age, years (mean ± SD)	41.81 ± 16.58	46.97 ± 17.81	U = 445.0	0.21
Male sex, n (%)	20 (64.5)	26 (74.3)	χ² = 0.74	0.39
Emergency surgery, n (%)	24 (77.4)	28 (80.0)	χ² = 0.07	0.80
Baseline wound area, cm² (mean ± SD)	46.35 ± 11.73	41.60 ± 14.08	U = 651.0	0.16

Primary outcome

The time to appearance of healthy granulation tissue and disappearance of slough was significantly shorter with indigenous NPWT than with conventional dressing (9.84 ± 1.19 vs 16.46 ± 4.20 days; mean difference 6.62 days, 95% CI 5.12-8.12; p < 0.001) (Table 2).

**Table 2:** Comparison of wound-healing and pain outcomes between indigenous NPWT and conventional dressing groups. Data are presented as mean ± SD, with between-group absolute differences expressed alongside their 95% CIs. Normality was assessed using the Shapiro-Wilk test; outcomes with nonnormal distributions were analyzed using the Mann-Whitney U test (U), and normally distributed outcomes using the Welch independent-samples t-test (t). NPWT, negative-pressure wound therapy; VAS, Visual Analog Scale

Outcome	Indigenous NPWT	Conventional	Difference (95% CI)	Test statistic	p-value
Time to healthy granulation, days	9.84 ± 1.19	16.46 ± 4.20	6.62 (5.12-8.12)	U = 8.0	<0.001
Number of dressing changes	2.10 ± 0.30	17.80 ± 4.69	15.70 (14.09-17.32)	U = 0.0	<0.001
Wound-area reduction at one week, %	54.2 ± 10.3	42.7 ± 14.9	11.47 (5.24-17.69)	U = 816.0	<0.001
Granulation rate, cm²/day	2.50 ± 0.58	1.00 ± 0.18	1.50 (1.28-1.72)	t = 13.82	<0.001
VAS at 24 hours	3.81 ± 0.75	6.03 ± 0.79	2.22 (1.84-2.60)	U = 30.0	<0.001
VAS at 48 hours	2.81 ± 0.75	5.03 ± 0.79	2.22 (1.84-2.60)	U = 30.0	<0.001
VAS at seven days	1.81 ± 0.75	4.03 ± 0.79	2.22 (1.84-2.60)	U = 30.0	<0.001

Secondary outcomes

The frequency of dressing changes required was substantially decreased in the NPWT group (2.10 ± 0.30 vs 17.80 ± 4.69; p < 0.001) and patients achieved greater wound-area reduction at one week (54.2% ± 10.3% vs 42.7% ± 14.9%; p < 0.001) and a faster rate of granulation-tissue formation (2.50 ± 0.58 vs 1.00 ± 0.18 cm²/day; p < 0.001). Pain scores were consistently lower in the NPWT group at 24 hours (3.81 ± 0.75 vs 6.03 ± 0.79), 48 hours (2.81 ± 0.75 vs 5.03 ± 0.79), and seven days (1.81 ± 0.75 vs 4.03 ± 0.79) (all p < 0.001) (Table 2).

Additional debridement and complications

Fewer patients in the NPWT group required additional debridement (7/31, 22.6%) than in the conventional group (22/35, 62.9%; p = 0.001). Complications were infrequent overall (5/66) and did not differ significantly between groups (2/31, 6.5% vs 3/35, 8.6%; p = 1.00) (Table 3).

**Table 3:** Categorical clinical outcomes by treatment group. Complications comprised bleeding, infection, and maceration. Groups were compared with Fisher’s exact test. NPWT, negative-pressure wound therapy.

Outcome	Indigenous NPWT (n = 31)	Conventional (n = 35)	p-value
Additional debridement, n (%)	7 (22.6)	22 (62.9)	0.001
Complications, n (%)	2 (6.5)	3 (8.6)	1.00

**Figure 1:**
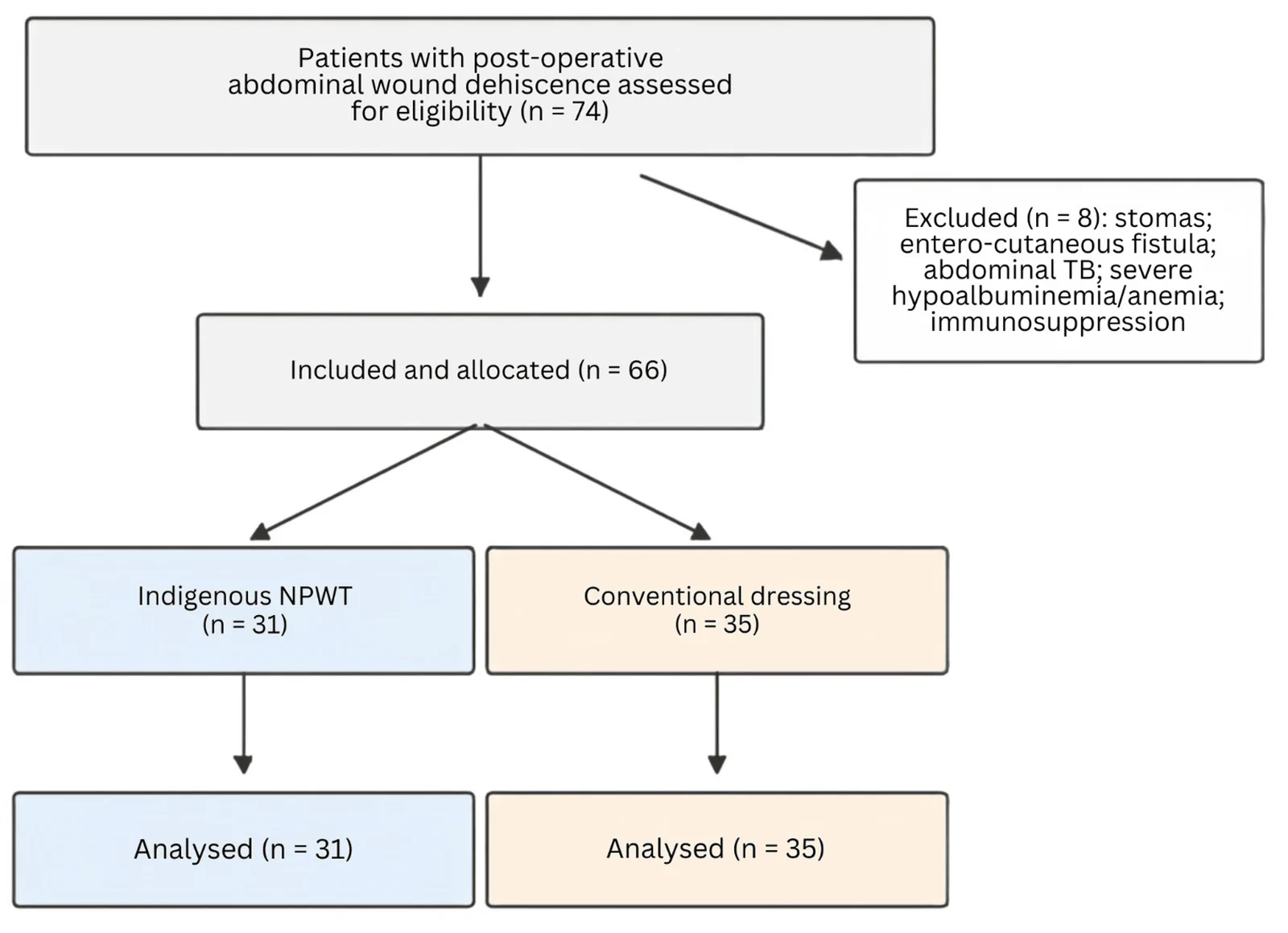
Participant flow diagram according to the STROBE guidelines. Patients with postoperative abdominal wound dehiscence were assessed for eligibility and allocated to indigenous NPWT (n = 31) or conventional saline-gauze dressing (n = 35); all were analyzed. NPWT, negative-pressure wound therapy; STROBE, STrengthening the Reporting of OBservational studies in Epidemiology

The participant flow is summarized in Figure 1.

## Discussion

In this single-center, prospective, comparative study of 66 patients with postoperative abdominal wound dehiscence, an indigenous, low-cost NPWT system was associated with faster appearance of healthy granulation tissue, fewer dressing changes, greater reduction in wound area during the early phase of healing, enhanced granulation-tissue formation, and reduced pain scores than conventional saline-gauze dressing. The baseline characteristics were comparable between the two study groups, and complication rates were low and similar, supporting the feasibility and tolerability of the indigenous system.

These findings are consistent with the broader NPWT literature. NPWT has repeatedly been shown to accelerate granulation and improve wound-healing metrics compared with conventional dressings across wound types [20], and NPWT-based TAC achieves high fascial-closure rates in the open abdomen [7,8]. Our results also align with reports from LMIC settings indicating that indigenous NPWT can reproduce the wound-healing benefits of commercial devices at a small fraction of the cost [12,13], and with comparative work showing shorter healing times for NPWT than conventional dressing [14]. The mechanistic basis, such as reduced edema, improved perfusion, exudate removal, and mechanical wound-edge approximation, offers a plausible explanation for the faster granulation and larger early area reduction observed here [10].

The marked reduction in the number of dressing changes has practical implications in resource-limited settings, where it translates into fewer procedures, reduced nursing workload, and potentially lower consumable use, alongside the lower pain scores reported by patients. Although a formal cost-effectiveness analysis was not performed, the system is assembled entirely from inexpensive, widely available materials (open-cell foam, standard suction tubing, an adhesive drape, and existing wall suction) and therefore represents a potentially low-cost alternative that can be deployed where commercial NPWT is unaffordable or unavailable [11].

Strengths of this study include its prospective design, the balanced baseline characteristics of the two groups, and the evaluation of multiple clinically relevant outcomes with complete data. Several limitations should be acknowledged. Allocation followed the admitting unit’s routine practice rather than randomization, introducing potential selection bias, unit-level confounding, and differences in co-interventions. Baseline comparability was demonstrated for a limited set of variables only; major prognostic factors, including nutritional status, diabetes, wound classification, and contamination, were not measured or adjusted for, so balance across all confounders cannot be assumed. The cohort may also encompass differing severities of dehiscence, introducing clinical heterogeneity. The number of dressing changes was partly determined by the prespecified NPWT inspection schedule (days 5 and 8) vs regular conventional changes and therefore reflects protocol design as well as any treatment effect; similarly, the timing of granulation assessment differed between arms. Wound area was measured by ruler-based length × breadth rather than formal planimetry or digital software, which may reduce measurement precision. Pain was analyzed by separate between-group tests at each time point rather than a repeated-measures model, and VAS is a subjective measure. Follow-up focused on early wound-healing parameters; definitive endpoints like time to secondary suturing, fascial-closure rate, length of stay, incisional hernia, and recurrence were not assessed. No formal cost or cost-effectiveness analysis was performed. Finally, the study was single-center with a comparatively small sample size and was not powered to compare complication rates; the absence of a significant difference in complications should not be interpreted as evidence of equivalent safety, and outcome assessment was not blinded.

Future work should include adequately powered, multicenter randomized controlled trials comparing indigenous and commercial NPWT with conventional dressing, incorporating long-term closure outcomes and formal cost-effectiveness analysis, to establish the role of low-cost NPWT in the management of the open abdomen in resource-limited settings.

## Conclusions

In this single-center, prospective, comparative study, indigenous NPWT was associated with faster granulation and improvement in early wound-healing parameters, fewer dressing changes, greater early wound-area reduction, and less pain than conventional saline-gauze dressing in postoperative abdominal wound dehiscence, with a comparable and low complication rate. As a low-cost, locally assembled alternative to commercial devices, indigenous NPWT is a practical and promising option for resource-limited settings. These findings should be validated in adequately powered, multicenter randomized controlled trials with long-term follow-up, including evaluation of fascial closure outcomes and cost-effectiveness.
